# Therapeutic Potential of Resveratrol in Cancer and Neurodegenerative Disorders: A Current Review

**DOI:** 10.1002/biof.70080

**Published:** 2026-01-26

**Authors:** Kenly Wuputra, Chia‐Che Ku, Ying‐Chu Lin, Yi‐Chun Tsai, Deng‐Chyang Wu, Yukio Mitsui, Maki Satou, Yuuki Tanaka, Shigeo Saito, Kazunari K. Yokoyama

**Affiliations:** ^1^ Graduate Institute of Medicine, Department of Medicine Kaohsiung Medical University Kaohsiung Taiwan; ^2^ Regenerative Medicine and Cell Research Center Kaohsiung Medical University Kaohsiung Taiwan; ^3^ Cell Therapy and Research Center Kaohsiung Medical University Hospital Kaohsiung Taiwan; ^4^ Division of Nephrology, Department of Internal Medicine Kaohsiung Medical University Hospital Kaohsiung Taiwan; ^5^ School of Dentistry Kaohsiung Medical University Kaohsiung Taiwan; ^6^ Division of Nephrology, Department of Internal Medicine Kaohsiung Medical University CiJin Hospital Kaohsiung Taiwan; ^7^ Division of Gastroenterology, Department of Internal Medicine Kaohsiung Medical University Hospital Kaohsiung Taiwan; ^8^ Research Institute Horus Co. Ltd Saitama Japan; ^9^ Saito Laboratory of Cell Technology Tochigi Japan

**Keywords:** anticancer, anti‐neuronal disease, antioxidant, reactive oxygen species, stem cell regeneration, therapeutic compounds

## Abstract

Resveratrol (RSV; 3,5,4′‐trihydroxy‐trans‐stilbene) is a natural polyphenolic compound with notable antioxidant, anti‐inflammatory, and immunomodulatory properties. It has been investigated for therapeutic applications in cardiovascular disease, cancer, and neurodegenerative disorders. This review emphasizes the potential of RSV in oncology and neuroprotection, synthesizing evidence from systematic database searches and experimental studies. Despite promising biological activities, RSV is limited by poor stability, low aqueous solubility, rapid metabolism, and restricted bioavailability, necessitating improved delivery strategies such as nanoencapsulation, nanocrystals, prodrugs, and structural analogues. Mechanistically, RSV exerts anticancer and neuroprotective effects through modulation of p53, STAT3, NF‐*κ*B, and mitochondrial‐mediated apoptosis. Its antioxidant actions involve regulation of reactive oxygen species (ROS), activation of NRF2, AMPK signaling, and SIRT1. RSV and related antioxidants act on multiple molecular pathways, including TP53, *β*‐catenin, STAT3, NF‐*κ*B, NRF2–AMPK, PI3K/AKT, and SIRT1, to regulate inflammation and cell death. The balance between oxidative and antioxidative processes is critical for therapeutic efficacy. Notably, RSV‐induced ROS‐mediated cell death, particularly in the context of TP53 mutations, represents a promising target for future interventions. Overall, RSV demonstrates multi‐target potential for cancer and neurodegenerative disease therapy, though optimization of its pharmacological profile remains essential.

## Graphical abstract

Resveratrol induces the expressions of TP53, NRF2, and JDP2 axis in cancer cells to control ROS balance in mitochondria. The antioxidation responses were induced through SRIT1/3, HDAC, STAT3, TGF*β*, and so on, to trigger the inflammation, and NFkB response to lead to the cell death of cancers or neuro‐damaged cells.

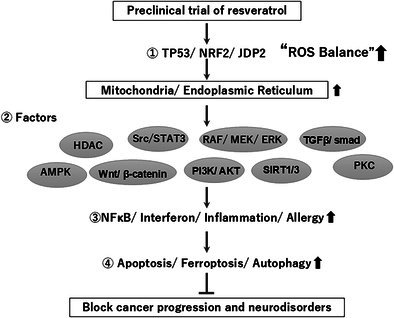

**Abbreviations**
AD: Alzheimer's disease
AHR: aryl hydrocarbon receptor
AMPK: 5′‐adenosine monophosphate‐activated protein kinase
ARF: alternate open reading frame
Bax: Bcl‐2‐associated X protein
Bcl2: B‐cell lymphoma 2
BMSC: bone marrow‐derived mesenchymal stem cells
CA: cinnamaldehyde
ERK1/2: extracellular signal‐related kinase 1/2
Exos: exosomes
GC: gastric cancer
GFAP: glial fibrillary acidic protein
GPX4: Glutathione peroxidase 4
HER2: epidermal growth factor receptor 2
NEDD4L: neural precursor cell expressed developmentally downregulated gene 4‐like
NOS: nitric oxide synthase
NRF2: nuclear factor erythroid 2‐related factor 2
NSC: neural stem cells
PAH: perillaldehyde
PD: Parkinson's disease
PI3K: phosphoinositide 3‐kinase
ROS: reactive oxygen species
SFN: sulforaphane
SIRT: sirtuin
TNBC: triple‐negative breast cancer
tRSV: *trans‐*resveratrol

## 1 Introduction

**FIGURE 1:**
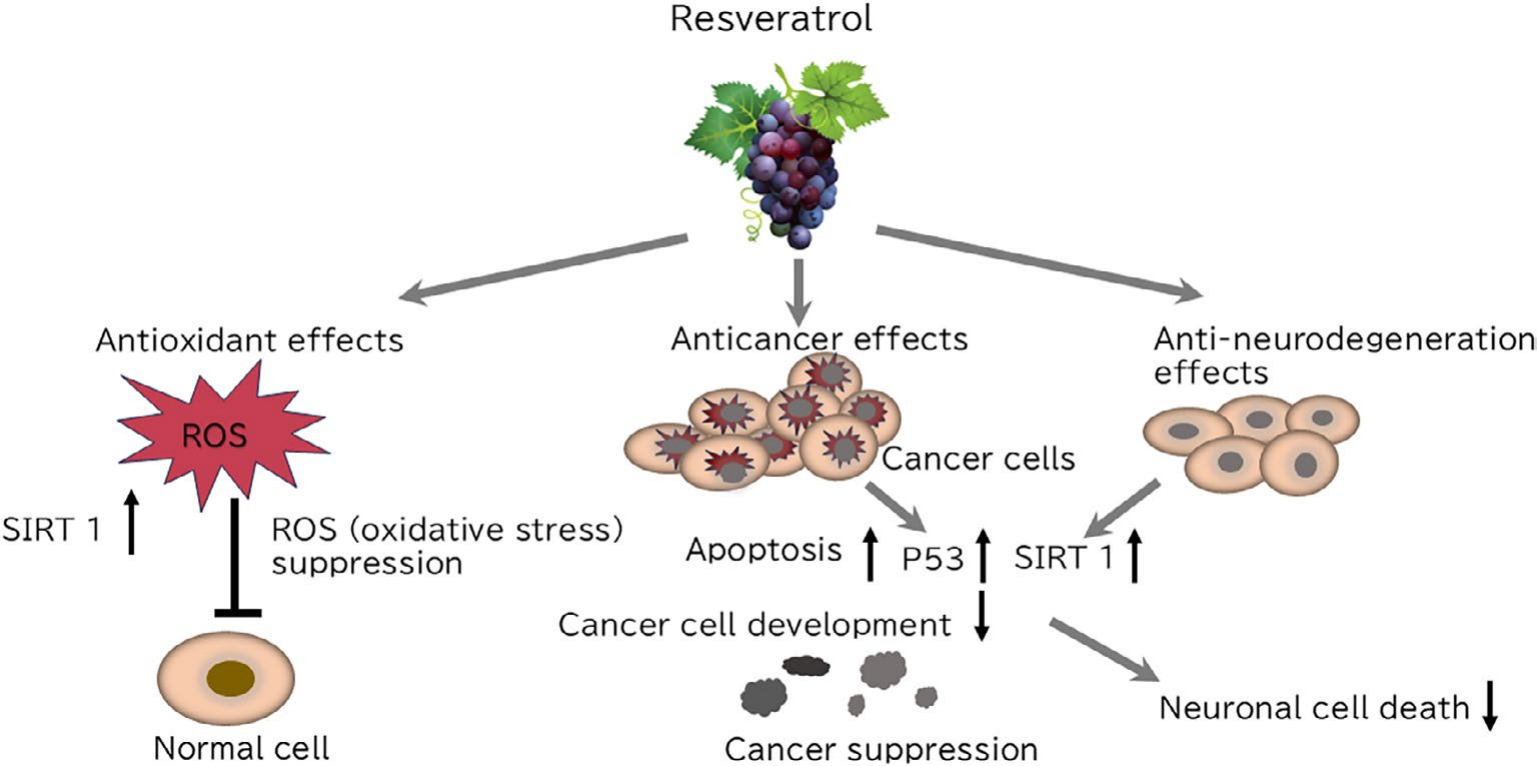
Schematic model of possible effects of Resveratrol to normal and cancer cells. In normal cells, resveratrol (RSV) induces antioxidant effects by increasing SIRT1 and suppression of ROS generation, and in cancer cells, RSV induces enhanced expression of p53, SRIT1 and increases apoptosis for cancer suppression to inhibit development of cancer cells and in neural protection RSV inhibits the neural death of neurons.

Resveratrol (3,5,4′‐trihydroxy‐*trans*‐stilbene, RSV) is a polyphenol compound found in some vegetables and fruits, including grape skins and seeds, berries, peanuts, and tea [1, 2]. In addition, RSV is a constituent of red wine [2, 3]. RSV possesses three hydroxyl groups attached to the aromatic rings, which are crucial for its biological activities, including antioxidant activity. Furthermore, this compound has anti‐inflammatory and immune modulatory properties, potentially protecting against various diseases, including cancer, cardiovascular disorders, and neurodegenerative conditions [3, 4, 5, 6], and extending the lifespan in yeast, nematodes, and fish [1, 2, 7, 8]. In addition, Baur et al. [9] reported that RSV prolonged lifespan in mice, a finding that garnered significant public and scientific interest. However, further investigation raised concerns about the validity of these findings, revealing serious flaws in many studies that attributed lifespan extension to the activation of sirtuin (SIRT) proteins by resveratrol. Instead, other research suggested that these observed longevity effects were mediated by alternative genetic mechanisms, independent of SIRTs [10, 11, 12]. RSV has beneficial properties, including anticancer [13, 14], and antioxidant affects [15, 16] such as developments of stem cells [17] and neuron functions [13, 18] (Figure 1).

In the case of human patients, by reducing inflammation through its suppression of nuclear factor kappa B (NF‐*κ*B) signaling and inhibition of Janus kinase/signal transducer and activator of transcription (STAT) pathway phosphorylation, RSV can impact immune and cellular responses to infections and stimuli [19, 20]. NF‐*κ*B, a transcription factor that promotes inflammation, played a role in the development and progression of tumors when abnormally activated. NF‐*κ*B targets genes that promote cancer cell proliferation, metastasis, inflammation, invasion, and angiogenesis [21]. Thus, inhibiting NF‐*κ*B expression is a promising strategy for cancer treatment. Because the concentration of RSV varies significantly between dietary sources and even within the same source, it is difficult to estimate the daily intake per person [22, 23]. Supplemental RSV is commercially available in tablets or capsules, with doses ranging from 1 to 500 mg [24]. Rapid absorption, poor bioavailability, and low water solubility are the main limitations for the oral intake of resveratrol [25, 26, 27].

**FIGURE 2:**
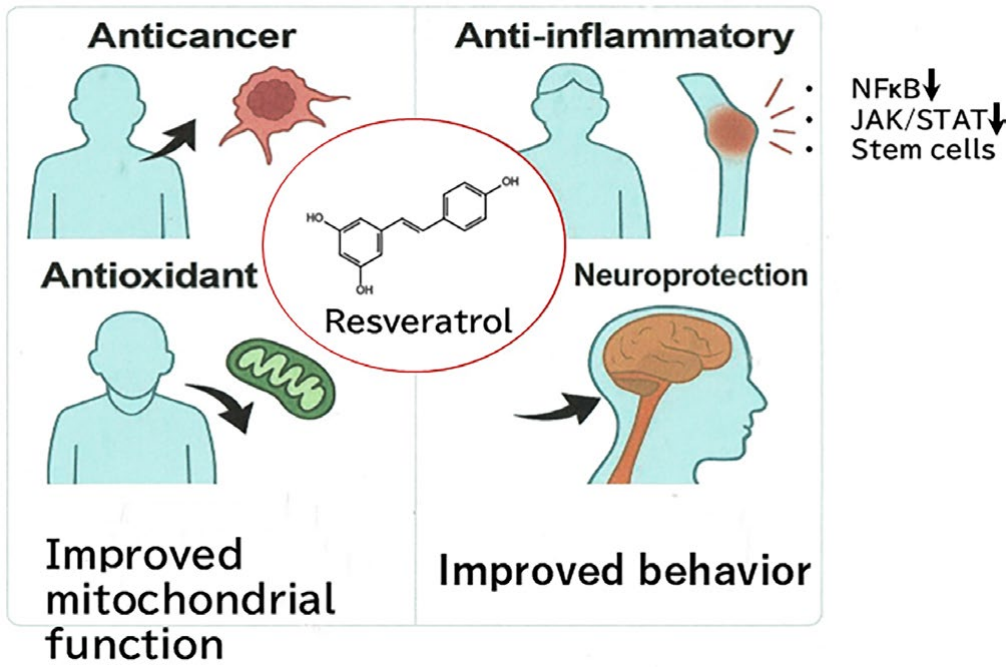
Schematic model of preclinical research of Resveratrol for human patients. The resveratrol (RSV) is used for the preclinical applications of anticancer, anti‐inflammation, neuroprotection, and antioxidant response to improve the mitochondrial function.

As we have discussed, the efficacy of RSV in promoting human health and treating diseases has shown mixed results in studies. In this review, we evaluate the current understanding of RSV's effects on anticancer activity, antioxidative properties in mitochondria function, anti‐inflammation, and neuroprotection to improve the behavior as therapeutic perspective (Figure 2). In addition, we summarised the biomolecular mechanisms of RSV in the context of advancing regenerative medicine.

## 2 Oxidative STESS and Antioxidation Reactions

Oxidative stress in cells can be generated by reactive oxygen species (ROS) and nitric oxide synthase (NOS).

ROS can be divided into two main categories: (i) oxygen molecules with an unpaired electron (free radicals), which include superoxide anion radicals, hydroxyl radicals, lipid peroxyl radicals, and nitric oxide radicals; and (ii) oxygen molecules in an excited state, represented by singlet oxygen. Notably, superoxide anion radicals were generated by various enzymatic reactions, including those catalyzed by xanthine oxidase and NADPH oxidase, in addition to being a byproduct of the respiratory chain [28].

### 2.1 Endogenous Factors

Genetic and metabolic factors, along with variations in hormone levels, have key effects on the balance of oxidation and reduction within the body, significantly impacting how an individual experiences aging. The body generates its own antioxidants, such as superoxide dismutase and glutathione, which serve to combat oxidative stress and protect against cellular harm. Telomere length, maintained by the enzyme telomerase, progressively shortens with cell division and decreases over time. Endogenous production of ROS can result in oxidative stress, leading to cellular damage. Persistent inflammation is a key characteristic of aging and can be affected by internal factors [29].

### 2.2 Exogenous Factors

ROS can be triggered by various external factors, including pollutants, heavy metals, tobacco smoke, pharmaceuticals, xenobiotics, microplastics, and radiation. Ionizing radiation can produce harmful intermediates by interacting with water, a phenomenon known as radiolysis. Given that water comprises 55%–60% of the human body, the likelihood of radiolysis occurring in the presence of ionizing radiation is significant. During this process, water loses an electron, becoming highly reactive. This starts a three‐step chain reaction, transforming water into hydroxyl radicals, hydrogen peroxide, superoxide radicals, and ultimately oxygen. The hydroxyl radical is highly reactive and quickly extracts electrons from nearby molecules, converting them into free radicals and perpetuating a chain reaction. By contrast, hydrogen peroxide is more detrimental to DNA than the hydroxyl radical, as its lower reactivity allows it to penetrate the cell nucleus and interact with essential macromolecules like DNA.

Dietary intake is another critical exogenous factor. Certain foods and supplements, particularly those rich in antioxidants and polyphenols, can aid in mitigating the effects of stress. Exposure to ultraviolet radiation, pollution, and smoking can hasten the aging process. Factors such as diet, physical activity, stress management, and sleep patterns can significantly affect survival. Incorporating antioxidants through diet or supplements can help alleviate oxidative stress. Some externally sourced bioactive peptides have shown potential antiaging effects. Meanwhile, advanced glycation end products, commonly found in processed foods, might influence aging and longevity [30].

## 3 Anticancer Properties

Genetic, epigenetic, and microenvironmental factors severely cause normal cells to transform into cancer‐initiating cells [31]. In normal tissues and organs, the balance between cell division and apoptosis is generally maintained to ensure tissue integrity. However, when this balance is disrupted by excessive cell proliferation and insufficient cell death, cells enter the initiation phase of malignant cancer. This principle forms the foundation of the widely accepted pathways of tumorigenic initiation and progression. Cancer initiation can result from epigenetic reprogramming that induces tumorigenic enhancer reactivation in both somatic and cancer cells [32].

**FIGURE 3:**
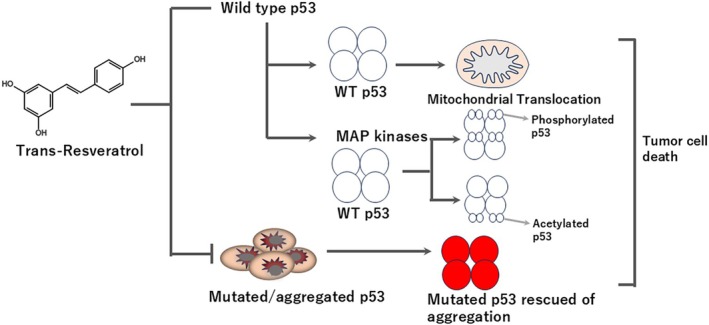
Possible models of cellular functions of resveratrol for TP53 normal and mutated cells. The co‐localization assays to detect TP53 aggregates in some breast cnacer cells expressing the p53 mutant R248Q and ther p53 hot‐spot mutants were performed and found that hot‐spot mutants have a greater tendency to aggregate than wild‐type p53 [33, 34]. Resveratrol has been shown to inhibit amyloid aggregation by binding to several amyloidogenic proteins like transthyretin, islet amyloid polypeptide, amyloid beta peptide, and alpha‐synuclein. The part of the anti‐tumoral effects of resveratrol might be related to the inhibition of p53 aggregation. The involvement of the p53 pathway in the effects triggered by resveratrol in cancer cells is summarized in this figure. The cancer cells with wild type p53 were treated by resveratrol, triggered to translocate into the mitochondria or modified by phosphorylation or acetylation during the cell cycle. Then, these cells underwent cell death by apoptosis, necrosis, ferroptosis, and so on. RSV inhibited the aggrgation of mutated TP53 and then rescued cells of the aggregation are let to the cell death [35].

p53 is a central tumor suppressor essential for cancer prevention. Wild type p53 halts tumor growth by inducing cell cycle arrest or apoptosis (Figure 3). Acting as a transcription factor, it regulates target genes that drive growth arrest, senescence, differentiation, and cell death under stress conditions such as DNA damage, oncogene activation, hypoxia, and telomere erosion [36, 37, 38]. The p53 pathway is highly sensitive to minimal DNA damage, enabling early detection of genetic lesions. In addition, the post‐translational modifications of p53 can provoke inappropriate cell cycle arrest and cell death. Mutant p53 aggregates exert nonspecific cytotoxicity and can themselves induce tumor cell death [36, 39]. Because wild type p53 overexpression strongly promotes apoptosis, cancer cells often disable p53 or its downstream pathways during oncogenesis. The pluripotency factor c‐Myc, widely expressed in cancer, accelerates progression in the absence of p53.

**FIGURE 4:**
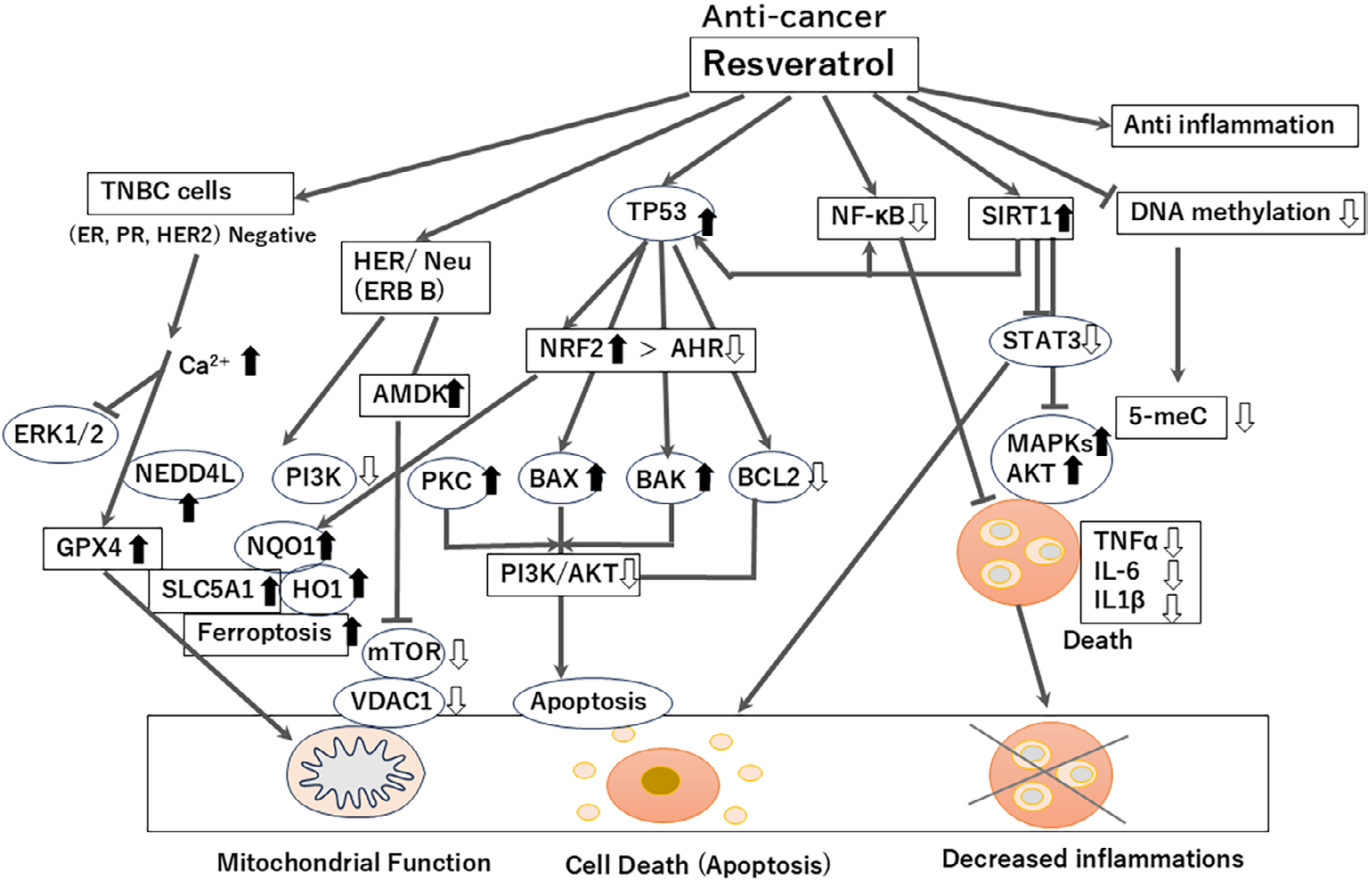
Potential molecular targets of resveratrol in signaling pathways for anti‐cancer effects. Resveratrol targets several molecules included aryl hydrocarbon receptor (AhR), nuclear factor erythroid 2‐related factor 2 (Nrf2), cyclooxygenase (COX), silent information regulator 1 (SIRT1), activator protein‐1 (AP‐1), nuclear factor kappa B (NF‐*κ*B), adenosine monophosphate‐activated protein kinase (AMPK), tumor protein p53 (TP53), estrogen receptor (ER), solute carrier family 7 member 11 (SLC7a11), glutathione peroxidase 4 (GPX4), NADPH oxidase, quinone oxidoreductase 1 (NQO1), heme oxygenase 1 (HO‐1), phosphoinositide 3‐kinase (PI3K)/AKT, mammalian target of rapamycin (mTOR), voltage‐dependent anion channel 1 (VDAC1), NLR family pyrin domain containing 3 (NLRP3), STAT3, transcription factor EB (TFEB), tumor necrosis factor *α* (TNF*α*), interleukin 1 beta (IL‐1*β*), IL‐6, and COX1/2. These targets play a crucial role in the anti‐inflammatory, antioxidative, neural protection, mitochondrial function, cell death, and analgesic effects of resveratrol. →: Promotion; ⊥: Inhibition. Filled arrow; increasing; white arrow; decreasing.

Resveratrol (RSV) has been shown to modulate p53 activity, thereby reinforcing its tumor‐suppressive functions. RSV can enhance p53‐dependent transcriptional programs that promote apoptosis, senescence, and cell cycle arrest, while also counteracting the deleterious effects of mutant p53 aggregation. Through these mechanisms, RSV restores p53 pathway integrity and sensitizes cancer cells to stress‐induced death. This dual capacity—supporting wild‐type p53 function and mitigating mutant p53 toxicity—positions RSV as a promising agent for targeting p53 dysregulation, a hallmark of many cancers. Resveratrol (RSV) demonstrates anticancer activity across multiple tumor types, including lung, ocular, pancreatic, breast, colorectal, and liver cancers [40, 41, 42, 43, 44, 45, 46]. However, the fundamental molecular mechanisms underlying its effects remain only partly understood. The possible targets of RSV were investigated and summarized (Figure 4).

RSV modulated the p53‐mediated pathway to induce apoptosis because it inhibited the viability of colorectal cancer cells [45]. RSV enhanced the expression of p53 and its target genes, including B‐cell lymphoma 2‐associated X protein (Bax) and p53‐upregulated modulator of apoptosis, which are critical mediators of p53‐dependent apoptosis in cancer cells.

To investigate the anticancer properties of RSV, various human cancer cell lines and animal models are commonly employed. Previous studies have shown that inhibition of protein kinase C isoforms by resveratrol was associated with decreased cell proliferation and the induction of apoptosis in cancer cell models, including human gastric cancer and prostate cancer cells [47, 48]. RSV treatment has also been shown to decrease viability and increase apoptosis in A549 human lung cancer cells, accompanied by the upregulation of BCL‐2‐antagonist/killer 1 (BAK) expression and cleaved caspase‐3, and downregulation of BCL‐2. The resveratrol analogue, *O*‐benzyl‐substituted 1,3‐diphenylpropane YI‐12, effectively inhibited the initiation and growth of cancer stem cells by targeting ERB B2 (also known as human epidermal growth factor receptor 2 [HER2]), a protein that regulates the phosphoinositide 3‐kinase (PI3K) signaling pathway in lung cancer cells [49].

In addition, RSV treatment activated p53 expression levels in a dose‐dependent manner (0–100 μM). In JJ012 chondrosarcoma tumor cells, resveratrol suppressed NF‐*κ*B signaling, leading to apoptotic cell death [50]. Furthermore, RSV influenced the level of DNA methylation by decreasing the activity of DNA methyltransferase enzymes and altering DNA methylation patterns by reducing 5‐methylcytosine levels. RSV also impacted inflammatory reactions by increasing activity in the SIRT1 and NF‐*κ*B pathways [51, 52, 53, 54].

Moreover, in vivo xenograft studies have revealed a marked reduction in tumor volume and dose‐dependent increases in SIRT1 and cleaved caspase‐3 expression [55]. RSV treatment has also been shown to induce apoptosis and inhibit cell growth, while also influencing STAT3 by activating SIRT1 in SW1353 chondrosarcoma cells [56].

Notably, RSV upregulated cleaved caspase‐3, SIRT1, and BAX, while downregulating BCL‐2 and STAT3 phosphorylation. These findings suggest that resveratrol modulates STAT3 phosphorylation via SIRT1 activation in SW1353 tumor cells in a dose‐dependent (0–100 μM) manner. RSV, known for its anticancer properties, also inhibited tumor necrosis factor (TNF), a cytokine involved in the activation of immune cells, cell differentiation, cell migration, and angiogenesis [57, 58]. Another key target of RSV is voltage‐dependent anion channel 1 (VDAC1), a protein crucial for apoptosis and mitochondrial function. VDAC1 facilitated the release of pro‐apoptotic proteins from the mitochondrial intermembrane space into the cytoplasm, promoting apoptosis in cancer cells [59, 60, 61].

More recently, Raviv et al. [58] showed that RSV increases VDAC1 expression, inducing apoptotic cell death in cancer cells. In general, apoptotic stimuli such as cisplatin, selenite, hydrogen peroxide, ultraviolet light, and other stressors triggered the overexpression of VDAC1, leading to its increased oligomerization [62]. The formation of VDAC1 oligomers created large channels that facilitated the release of pro‐apoptotic proteins, ultimately triggering cell death. In addition, RSV increased intracellular calcium and ROS and detached hexokinase from VDAC1 [59]. However, the poor solubility of RSV necessitated the development of novel delivery systems to enhance its bioavailability. Various strategies have been explored using liposomes, dendrimers, nanoparticles, and other carriers [63, 64, 65, 66, 67].

## 4 TP53 AS RSV Therapeutic Target (see Figures 3 and 4)

TP53‐mediated apoptosis occurs primarily through the transcriptional activation of its target genes, whereas TP53‐independent apoptosis is driven mainly by direct interactions of TP53 with anti‐apoptotic or pro‐apoptotic proteins such as NOXA, PUMA, and TP53AIP1. In addition, TP53 induces the transcription of pro‐apoptotic members of the BCL2 gene family, including BAX and BAK, which facilitate the release of cytochrome c into the cytoplasm. Cytochrome c then binds to apoptotic protease‐activating factor 1 (APAF‐1), leading to the oligomerization of the APAF‐1/caspase‐9 complex (apoptosome). This complex recruits pro‐caspase‐9 and subsequently activates effector caspases. Furthermore, TP53 can promote apoptosis through the activation of death receptors such as FAS, DR4, and DR5 [68, 69].

RSV‐induced TP53 activation and apoptosis have been shown to be mediated by mitogen‐activated protein (MAP) kinases. Liao et al. [70] demonstrated for the first time that RSV upregulated endogenous TP53 levels, particularly in its phosphorylated state, in epidermal JB6 cells—a well‐established model for studying tumor progression. Likewise, phosphorylated protein kinases, including ERKs, p38 kinase, and JNKs, increased in a time‐dependent manner following resveratrol treatment [71]. Ishimaru et al. reported that physical unfolding of the p53 core domain (p53C) led to the formation of distinct types of aggregates [72]. Furthermore, a small cognate double‐stranded DNA was shown to stabilize both p53C and full‐length p53, thereby rescuing aggregated and misfolded protein species [73].

In addition to these in vitro findings, Levy et al. [33] identified TP53 aggregates in archived breast cancer tissue samples expressing the TP53 mutant R248Q and other hot‐spot mutations. Their study revealed that hot‐spot mutants have a greater tendency to aggregate compared to wild‐type TP53. RSV has been shown to inhibit amyloid aggregation by binding to several amyloidogenic proteins, including transthyretin, islet amyloid polypeptide (IAPP), amyloid‐*β* peptide, and *α*‐synuclein. Costa et al. [34] examined the interaction between RSV and p53, evaluating its effect on p53 amyloid aggregation. Their findings indicated that part of the anti‐tumoral activity of RSV may be attributed to its ability to inhibit TP53 aggregation. Collectively, these data highlight the involvement of the TP53 pathway in the anticancer effects elicited by RSV.

## 5 Nanoparticle Based RSV Delivery System for Function

**FIGURE 5:**
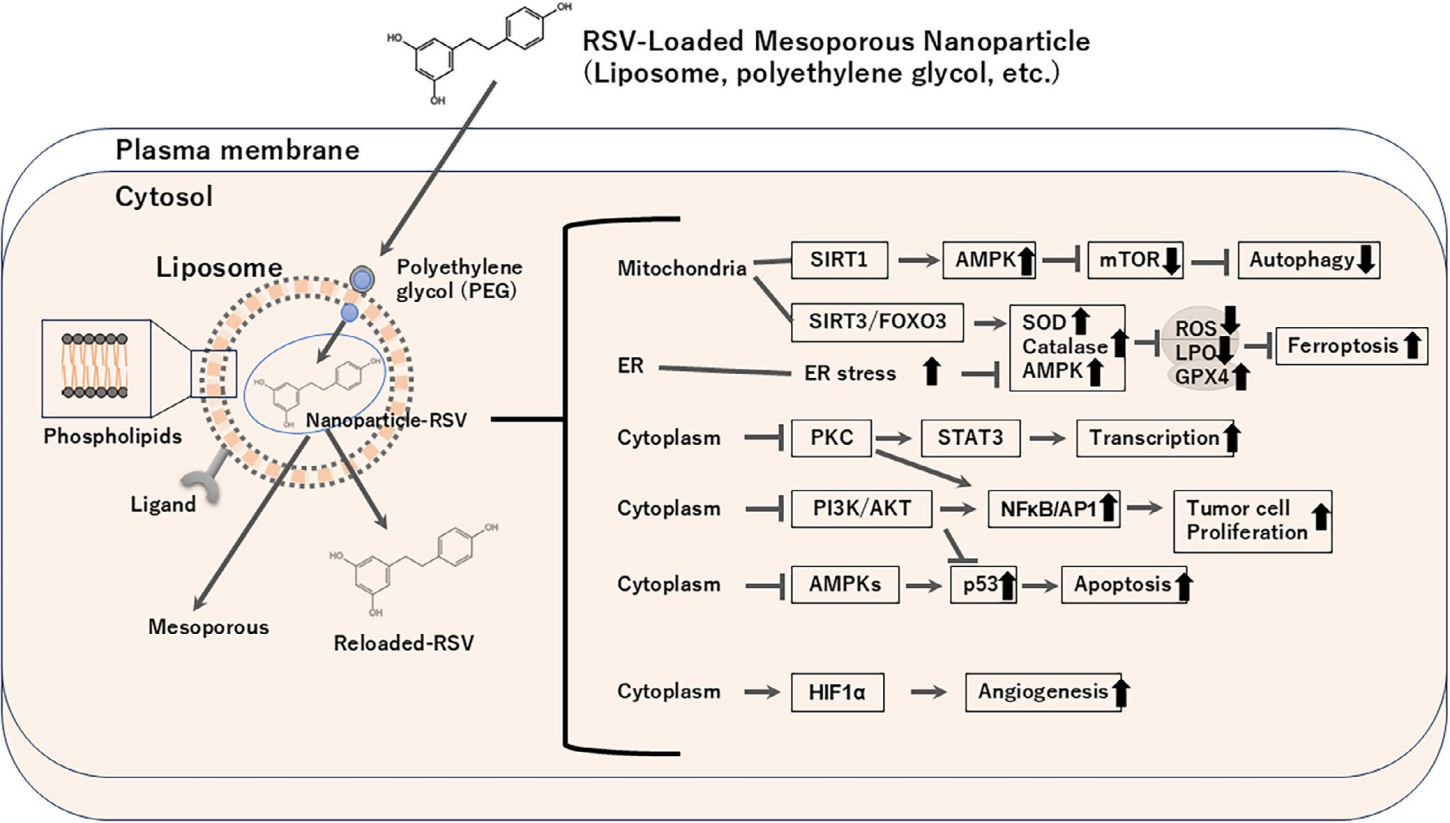
Overall mechanistic pathways of resveratrol in the cells. RSV loaded mesoporous nanoparticles were applied to the cells and then it transmitted to various signal pathways to control the functions such as autophagy, ferroptosis, transcription, apoptosis, angiogenesis and tumor cell proliferation. silent information regulator 1 (SIRT1), Adenosine monophosphate kinase (AMPK), mammalian target of rapamycin (mTOR), Superoxide dismutase (SOD), reactive oxygen species (ROS), Lipid peroxidase (LPO), glutathione peroxidase 4 (GPX4), protein kinase C (PKC), nuclear factor kappa B (NF‐*κ*B), activation factor 1 (AP1), and hypoxia inducing factor 1 alfa (HIF 1*α*).

In general, nanocarrier‐based drug delivery systems typically utilize particles of sizes smaller than 500 nm [74] (Figure 5). In one study, encapsulating RSV in poly (d, l‐lactic‐co‐glycolic acid) nanoparticles and combining it with free sunitinib, a tyrosine kinase inhibitor, resulted in a ninefold increase in RSV concentration and enhanced antitumor efficacy. Combining sunitinib and RSV at a ratio of 1:8 synergistically reduced the viability of HT‐29 colorectal cells. This synergy was confirmed by combination index values less than 1, based on their respective IC_50_ values of 14.4 and 110.9 μM [75, 76]. Triple‐negative breast cancer (TNBC), characterized by the absence of estrogen, progesterone, and HER2 receptors, is often associated with ineffective treatment. RSV inhibited the expression of glutathione peroxidase 4 (GPX4) protein by increasing neural precursor cell expressed developmentally downregulated gene 4‐like (NEDD4L)‐dependent ubiquitination. This occurs because RSV increased the interaction between NEDD4L and GPX4 by blocking extracellular signal‐related kinase 1/2 (ERK 1/2) and serum glucocorticoid‐regulated kinase 1/NEDD4L/GPX4 pathways in vitro and in vivo [77]. The mechanistic understanding by which resveratrol can induce ferroptosis in TNBC will provide a novel strategy for the treatment of this disease [78].

## 6 Antioxidant Properties Against ROS

ROS functions as a double‐edged sword in cell proliferation. Low levels of ROS acted as intracellular signaling molecules that were essential for cell progression, differentiation, and self‐renewal [79]. By contrast, excessive levels of ROS, which were byproducts of aerobic metabolism, can injure cells as revealed by DNA damage, cell senescence, or even cell death [80, 81].

The balance of ROS in cells is determined by the interplay between the production of oxidative stress and the cell's antioxidant defenses, such as the Nrf2‐mediated pathway [82]. This pathway also leads to improved mitochondrial function [60]. Our research group discovered that the aryl hydrocarbon receptor (AHR) plays a critical role in promoting ROS generation, cell spreading, invasion, and tumor regression in KRAS–p53 mutated pancreatic cancer cells in a mouse model [83]. Our study revealed that NRF2 was recruited to the AHR promoter via a complex formed with AHR itself, its nuclear translocator AHR nuclear translator, and the mediator Jun dimerization protein 2 (JDP2). These findings suggest that screening small molecules targeting the AHR–NRF2–JDP2 interaction could be a practical strategy for regulating oxidative stress and antioxidant responses involved in promoting detoxification, anti‐inflammatory, and anticancer effects [84, 85].

The antioxidant property of RSV was attributed to its ability to neutralize various free radicals (e.g., ROS, or reactive nitrogen species) [86]. RSV promoted the differentiation of MC3T3‐E1 cells in vitro and reduced bone loss in ovariectomized rats in vivo by activating the differentiation of normal bone marrow‐derived mesenchymal stem cells (BMSCs) [86, 87, 88]. In this context, Zhao et al. [89] reported that RSV attenuated senescence in BMSCs derived from aged rats and promoted their differentiation into the osteoblastic lineage. This effect was mediated by 5′‐adenosine monophosphate‐activated protein kinase (AMPK) activation, which suppressed ROS production. Thus, RSV enhanced the osteogenic differentiation of senescent BMSCs via the AMPK/ROS signaling pathway. AMPK served as a metabolic sensor that responded to oxidative stress by promoting metabolic reprogramming. Khamis et al. [90, 91] assessed the effects of *trans‐*resveratrol (T‐RSV) in improving biochemical and molecular alterations in obese Wistar male rats fed a high‐fat/high‐fructose diet. The authors found that T‐RSV restored the changes in carbohydrate–lipid metabolism, as well as oxidative stress, while simultaneously upregulating SIRT1, peroxisome proliferator‐activated receptor gamma coactivator 1 alpha, cytochrome c, and glucose transporter type 4 proteins. RSV is a promising treatment for obesity induced by high‐calorie diets by influencing molecular pathways and gene expression. RSV inhibited the formation of *Staphylococcus aureus* (S. aureus) biofilms by decreasing polysaccharide intracellular adhesion, extracellular DNA release, and ROS production. This discovery suggests the clinical application of RSV for treating *S. aureus* infections [92].

## 7 MSC Development and Neural Differentiation

RSV increased the proliferation and survival of neural stem cells (NSCs), which can improve cognitive function in tasks that depend on the hippocampus [93]. The effects of various concentrations of RSV on human MSC development have also been examined. At 0.1 μM, RSV promoted cell self‐renewal by inhibiting cellular senescence, whereas concentrations of more than 5 μM induced senescence, prolonged cell doubling time, and caused cell cycle arrest [13]. Whether RSV metabolites played significant biological roles is still unclear, but lower dosages should be further studied. In one study investigating different concentrations, 5 μM RSV increased both alkaline phosphatase activity and calcium deposition during osteogenic differentiation of periosteum‐derived MSCs compared with the untreated control [94]. Other studies have shown that higher concentrations of RSV (e.g., 25 μM) stimulated the osteogenic differentiation of MSCs [95, 96, 97]. Because the effects of RSV during differentiation are concentration‐dependent, further investigations are needed to determine the optimal dosage and effective administration for MSC‐based regenerative therapies.

Several types of stem cells can differentiate into spinal ganglion neurons, including endogenous auditory stem cells [98, 99], embryonic stem cells [100], NSCs [101], and dental pulp stem cells [102]. MSCs are also a promising source for neuronal differentiation [103]. Neuronal cells have been successfully derived from MSCs obtained from various tissues, including bone marrow [104], umbilical cord [105], cord blood [106], periodontal ligament [107], and both deciduous and permanent teeth [102]. RSV has been identified as a potent compound that enhanced both neuronal differentiation and neuroprotection [108]. Human MSCs treated with various concentrations of RSV have demonstrated increasing neuronal differentiation in a dose‐dependent manner by decreasing nestin expression and increasing *β* III tubulin, neuron‐specific enolase (NSE), neurogenin, and Mash1 as differentiation markers [109]. Similarly, RSV (15 μM) has been shown to enhance the expression of neuron‐specific markers such as nestin, musashi, and neurofilament M in human MSCs, contributing to their neuronal differentiation property [110]. Treatment of human MSCs with a high dose of RSV (30 μg/L) led to the detection of glial fibrillary acidic protein (GFAP) and glial markers, along with significant neuronal differentiation [102]. Immunohistochemical analysis confirmed the expression of GFAP, Nestin, and NSE in this study. As described above, RSV can play a pivotal role in the neuronal differentiation of MSCs. At a concentration of 10 μM, RSV (alone or with nerve growth factor) enhanced the phosphorylation of cAMP response element‐binding protein and the expression of neural markers in human cord blood stem cells via activation of protein kinase A, glycogen synthase kinase 3 beta, and ERK 1/2 [106]. Unpublished observations from our laboratory indicated that human amniotic stem cells [111], when cultured in the presence of 10 μM RSV, acquired neuronal phenotypes, suggesting a neuronal differentiation‐inducing effect of the compound under defined conditions.

Parkinson's disease (PD) was a progressive neurodegenerative disease primarily characterized by the loss of dopaminergic neurons in the substantia nigra. In addition, mitochondrial dysfunction, oxidative stress, and neuroinflammation drive the neurodegeneration in PD [112].

Exosomes (Exos) from human NSCs show promise in clinical applications for their neuroprotective effects through bioactive molecule transfer. RSV has been shown to increase the therapeutic potential of stem cell‐derived Exos. This finding highlighted the potential of human NSCs–Exo(s) as a novel therapeutic strategy for neurodegenerative diseases like PD, with RSV serving as a valuable enhancer of Exo efficacy [113]. RSV prevented excessive opening of the mitochondrial permeability transition pore in dopaminergic neurons, inhibited the abnormal aggregation of *α*‐synuclein (*α*‐syn) in mitochondria, and suppressed mitochondrial apoptosis signals. VDAC1 reversed the RSV‐induced decrease in *α*‐syn accumulation in mitochondria. Thus, VDAC1 might be a therapeutic target for PD because it played a crucial role in regulating mitochondrial function and apoptosis, both of which are implicated in PD pathology [112].

To assess the combination effects of RSV and coenzyme Q10 (CoQ10) on the neuronal differentiation of human MSCs, a medium supplemented with 2.5 μM RSV, 10 μM CoQ10, epidermal growth factor (20 ng/mL), and basic fibroblast growth factor (20 ng/mL) was used [113]. The results showed that combination treatment increased the expression of NSC cellular markers, which were previously damaged by the administration of 1‐methyl‐4‐phenylpyridinium, a common neurotoxin used in PD models. The authors concluded that the combination of RSV and CoQ10 enhanced cellular differentiation to NSCs. CoQ10, a cofactor in the electron transport chain, protected against neurodegeneration of the brain by promoting ATP generation and acting as an antioxidant to neutralize free radicals [114].

## 8 Clinical Efficacy of RSV in Neurogenerative Disorders

Numerous in vitro studies have shown that resveratrol exerted neuroprotective effects on cellular models of Alzheimer's disease (AD) [115, 116] and PD [112, 117]. AD is a progressive neurodegenerative disorder characterized by cognitive decline, memory loss, and neuronal dysfunction. It is driven by the accumulation of amyloid‐beta (A*β*) plaques, Tau protein hyperphosphorylation, oxidative stress, and neuroinflammation. In AD‐related models, RSV mitigated *β*‐amyloid‐induced cytotoxicity and promoted neuronal survival through SIRT1 activation and autophagy regulation [115, 116, 118].

**FIGURE 6:**
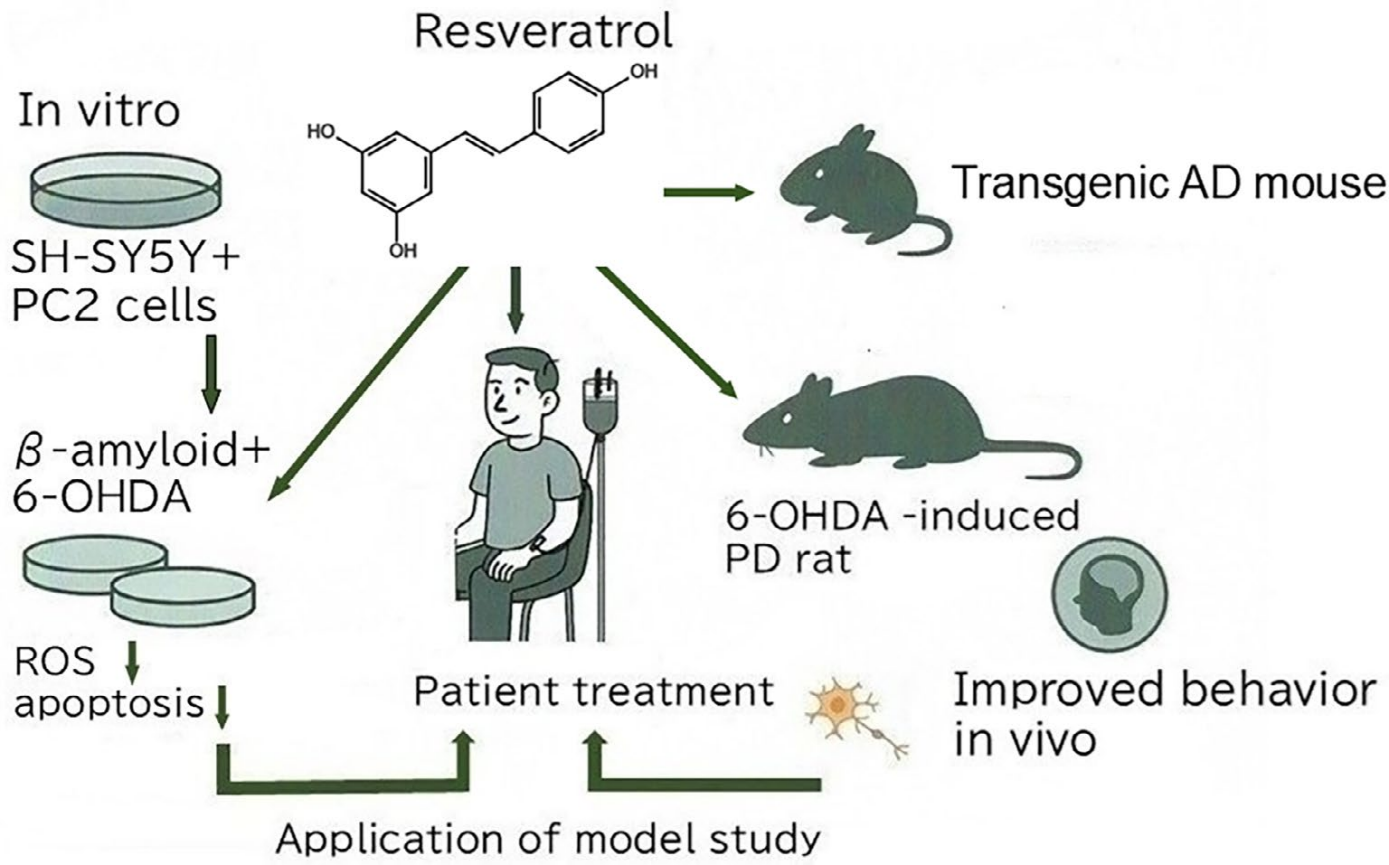
Neuroprotective effects of resveratrol on in vitro and in vivo models of AD and PD. SH‐SY5Y is a human cell line derived from a neuroblastoma, specifically a subclone of the SK‐N‐SH cell line (ATCC HTB‐11). The 6‐hydroxydopamine (6‐OHDA) rat model is widely used as an experimental tool for studying PD because injection of 6‐OHDA, a neurotoxin, destroys dopamine cells in one brain hemisphere.

Similarly, in PD models using dopaminergic SH‐SY5Y cells, RSV protected against 6‐hydroxydopamine (6‐OHDA)‐induced oxidative damage and apoptosis by modulating the Nrf2/heme oxygenase 1 (HO‐1) pathway [117, 119] (Figure 6). Co‐culture experiments with MSCs have revealed additive effects, such as enhanced neurotrophic factor secretion and neuronal regeneration, underscoring the synergistic therapeutic potential [118].

In vivo experiments using transgenic mouse models of AD and toxin‐induced PD models have confirmed the neuroprotective potential of resveratrol.

AD mouse models treated with RSV showed a reduced amyloid plaque burden, restored synaptic plasticity, and improved memory performance [118]. In 6‐OHDA‐induced PD rats, RSV administration led to the preservation of nigrostriatal dopaminergic neurons, reduced microglial activation, and improved motor coordination [119]. These findings confirm the bioactivity of RSV in modulating neuropathological hallmarks in vivo.

The potential of RSV in humans has been investigated in several clinical trials. For example, in a double‐blind, placebo‐controlled study involving patients with mild to moderate AD, daily oral supplementation of RSV (up to 1 g/day for 52 weeks) attenuated cognitive decline and preserved cerebrospinal fluid A*β*42 levels [120]. A separate pilot study reported improved cerebral blood flow and hippocampal functional connectivity after RSV supplementation in healthy older adults [121]. However, most other trials have highlighted challenges, including poor bioavailability, rapid metabolism, and interindividual variability in response [122]. AD is one of the major challenges facing modern medicine. Recently, dietary supplements have been recommended as a complementary strategy to prevent and treat this disease. Specific regimens such as curcumin (800 mg/day), omega‐3 fatty acids (2 g/day), resveratrol (600 mg/day), and other supplements like phosphatidylserine (300 mg/day), multi‐nutrient formulations, probiotics, vitamin E (2000 IU/day), and melatonin (3–10 mg/day) have demonstrated some benefits, although the degree of certainty regarding these effects varies [123].

Although certain supplements have been shown to mitigate the cognitive decline in AD, inconsistent findings and gaps in dosage and safety data highlight the need for rigorous, large‐scale preclinical and mechanistic studies to determine their efficacy.

Future research should focus on personalized, multimodal strategies integrating targeted supplementation, dietary patterns, and microbiota–gut–brain interactions for enhanced neuroprotection.

Despite promising results, the clinical practice of RSV faced several challenges. Its low bioavailability, due to rapid conjugation in the liver and intestine, limited its therapeutic application [124]. Formulation strategies such as nanoencapsulation and co‐delivery with bioenhancers were under investigation to improve systemic availability [125]. In addition, inconsistent outcomes across trials emphasized the need for standardized protocols, longer treatment duration, and biomarker‐guided patient selection [126]. Collectively, preclinical and limited clinical data suggested that RSV holds promise as a neuroprotective agent in neurodegenerative diseases. It exhibited multimodal effects on oxidative stress, protein aggregation, and inflammation [127]. Future studies should focus on improving delivery systems, elucidating their pharmacokinetic profile in target tissues, and identifying patient subgroups most likely to benefit from treatment [128]. Integrating RSV into combination therapies might also enhance efficacy [13].

RSVs pleiotropic mechanisms, ranging from antioxidative and anti‐inflammatory effects to the modulation of amyloidogenic and synuclein pathways, enabled a broad therapeutic spectrum [13, 18]. Although certain pathways like those involving SIRT1 activation or NF‐*κ*B inhibition might be consistently affected across patients or diseases, other pathways may vary depending on factors such as disease stage, cell type involved, and the presence of comorbidities [129]. This highlights the need for biomarker‐driven stratification in future trials. Moreover, the combinatorial potential of RSV with conventional therapies (e.g., cholinesterase inhibitors, dopamine agonists) remains underexplored [130]. Despite promising early findings, rigorous, large‐scale clinical trials were essential to confirm efficacy, establish optimal doses and ensure long‐term safety [97]. The integration of omics‐based approaches and systems biology models might further refine our understanding of its mechanistic landscape [131]. RSV is a compelling natural compound with multifaceted neuroprotective properties; its main dietary sources are apples, tomatoes, peanuts, and grapes [132, 133, 134].

## 9 Delivery Systems for RSV

As described above, RSV is known as a chemo‐preventive or cancer therapeutic agent and reduces the risk of cancer and various diseases. It mediates the regulation of angiogenesis, apoptosis, autophagy, and metastasis. However, several problems exist in the clinical application of RSV, including its low bioavailability, rapid metabolism, and cytotoxicity. Preclinical studies demonstrated the potential of RSV in the mitigation of degenerative musculoskeletal disorders, cardiovascular diseases, cancer progression, and neurological disorders. To overcome the abovementioned issues, different types of nanoparticles, liposomal carriers, and sustained‐release intravitreal implants have been developed for RSV [135, 136, 137, 138]. RSV‐loaded or ‐conjugated nanoparticles have a longer circulation time than free RSV molecules [139].

Pharmacokinetic studies demonstrated that the nanoencapsulated RSV had a longer half‐life [140] and increased bioavailability.

For example, the nanoparticles improved absorption of RSV into the gastrointestinal tract and protected against the first‐pass metabolic reaction in the liver [141]. Compared with free RSV molecules, nanoparticles enhanced RSV distribution in tumor tissue areas, which might be due to the enhanced permeability and retention in vessel structures of the tumor vasculature [139]. Nanoparticles protected RSV from rapid metabolic degradation and maintained the blood levels of RSV. In general, various strategies, such as drug‐release and stimulus‐responsive reactions, have been incorporated into RSV nano‐formulations to release RSV in response to specific internal or external stimuli [142] (Figure 5). The common stimuli‐responsive approaches are as follows: (i) pH‐responsive release, (ii) enzyme‐responsive release, (iii) temperature‐responsive release, (iv) redox‐responsive release, (v) light‐responsive release, (vi) magnetic‐responsive release, and (vii) ultrasound‐responsive release.

In pH‐sensitive nanoparticles, RSV was released at acidic pH in cancer microenvironments to enhance the drug delivery capacity to cancer cells while having a lower release in normal tissues [142]. In thermosensitive nanoparticles, the loaded RSV was released in response to local changes in temperature. Thus, hyperthermia can initiate RSV release efficiently. In addition, enzyme‐cleavable RSV in nanoparticles can be generated. The presence of matrix metalloproteinases in tumors enhanced the release frequency of RSV. Redox‐responsive nanoparticles can release RSV in response to high levels of reactive oxygen species (ROS) in tumor cells and their environments. Moreover, photo‐responsive materials were inserted into RSV nanoparticles, enabling light‐induced drug release. In addition, RSV‐loaded magnetic nanoparticles can be specifically delivered to their indicated target sites using external magnets. RSV‐loaded ultrasound‐responsive nanoparticles can be triggered to release their RSV at the specific target site using focused ultrasound waves. These stimulus‐activated methods offer precise control over drug‐release kinetics, thereby improving the targeting of RSV and minimizing side effects. The triggering choice depends upon the target's specific characteristics and the desired therapeutic outcome [142].

In the initial trial of RSV encapsulation, the sustained release of RSV using RSV‐loaded chitosan nanoparticles caused increased cell death compared with an equivalent dose of free RSV molecules [143]. Specifically, liposomes, polymerases, polymer constructions, and micelles were used as delivery methods for imaging. The inorganic nanoparticles exhibited material‐ and size‐dependent physicochemical characteristics, which are incomparable with those of conventional lipid‐ or polymer‐based nanoparticles [144].

Recently, RSV‐loaded magnetic niosome nanoparticles were also reported. Magnetic nanoparticles were loaded into niosomes using the thin‐film hydration method [145]. The cytotoxicity of RSV‐loaded magnetic niosome nanoparticles in the presence of an external magnetic field was higher than that of free RSV molecules, indicating increased cellular uptake of the encapsulated nanoparticles. In addition, RSV‐loaded magnetic nanoparticles triggered cell cycle arrest at the G0/G1 checkpoint more frequently than free RSV molecules. Compared with RSV‐treated cells, the expression levels of certain mRNAs, including BAX, BCL2, FAS, TP53, cyclin D, and hTERT, were significantly changed in cells treated with RSV‐loaded magnetic nanoparticles. Thus, the niosome nanoparticle approaches were efficient and produced higher solubility, improved bioavailability, and stability compared with control delivery of RSV [145].

Mesoporous silica nanoparticles loaded with RSV (MSN@Res) were also generated to improve the solubility and stability of RSV. MSN@Res suppressed the proliferation of hypertrophic scar fibroblasts by decreasing autophagy and increasing apoptosis and autosis cell death, which is an autophagy‐dependent, nonapoptotic, and nonnecrotic cell death process. Thus, the MSN@Res system is a novel drug delivery system characterized by excellent stability and high drug release capacity. It can inhibit protective autophagy of fibroblasts in hypoxic environments and induce apoptosis and autosis via the ROS‐mediated p38–mitogen‐activated protein kinase/hypoxia‐inducible factor‐1*α*/p53 signaling axis [146].

A lipid‐based auto‐emulsifying drug delivery system was also recently developed. In this system, RSV was solubilized in a hot liquid phase composed of lipids and surfactants, and this mixture was further adsorbed onto a powder composed of polysaccharides and sodium caseinate, along with inert excipients, and then compressed. The RSV‐containing tablets were characterized using differential scanning calorimetry and X‐ray diffraction. The digested formulation, with simulated gastric and enteric fluids, was dimensionally assessed using dynamic light scattering. Then, the calculations of the bioaccessible fraction, dissolution tests, and in vitro permeability experiments using colorectal adenocarcinoma cell monolayers were performed [146].

Poly (lactic‐co‐glycolic acid) polymer is known to be nontoxic, biodegradable, and biocompatible for delivering medication to the tumor location. The therapeutic efficacy of RSV, curcumin, and epigallocatechin gallate was evaluated using this polymer. In addition, poly (lactic‐co‐glycolic acid) nanoparticles can be modified by targeting molecules to the indicated cancer cells, thereby improving the effectiveness of phytochemicals in the indicated tumors [147].

The combination of nose‐to‐brain delivery via the intranasal route has garnered attention as a straightforward, noninvasive method for transporting nanoencapsulated RSV across the blood–brain barrier, resulting in enhanced effects and fewer side effects. Compared with the more traditional routes of administration, intranasal administration of RSV nanoformulations was less toxic, and more cost‐effective and efficient in the delivery across the blood–brain barrier. Therefore, this route represented a promising approach to managing central nervous system disorders [148]. Thus, new delivery systems for RSV have been developed to address the issue of low bioavailability.

## 10 Clinical Trials of RSV for Cancer, Neural Disease, and Other Diseases

Almost 200 studies of RSV performed over the last 20 years were grouped into at least 24 indications, including (i) cancer, (ii) neurological disorders, (iii) cardiovascular disease, (iv) diabetes, (v) nonalcoholic fatty liver disease (NAFLD), and (vi) metabolic syndrome and other diseases. Some recent clinical trials of RSV were listed (Table 1). Currently, no consensus on treatment regimens exists. However, the fact that RSV is generally well tolerated at doses of up to 1 g/day is well [168].

**TABLE 1:** Recent clinical trails of RSV (2024–2025).

2025 disease	Dose	Effect	References
Hypertension (HP) [IRCT201407078129N7] First trail 15/08/2024	Pre‐hypertension [90–99 mmHg] [120–137 mmHg] Stage I HP [140–159 mmHg] 2/day for 4 weeks (orally) or placebo 2/day for 4 weeks in a 2 × 2 crossover design	RSV was injected randomized crossover, double‐blind placebo‐control. Nitric oxide (NO) was higher in RES treated group than placebo treated group.	[149]
COVID‐19 patients [IRCT20111119008129N13]	750 mg/day orally or placebo for 20 days	C‐reactive protein (CCRP)↓ Fasting blood sugar (FBS)↓ Alkaline phosphatase↓ IL‐1*β*, TNF‐*α* ↓ White blood cells ↓ Platelet count↓ Lymphocyte↑in REV Patients > placebo patients	[150]
Alzheimer disease (AD)	Moderate AD RSV‐neuro protection →through SIRT‐1 →A*β* aggregation →tau phosphorylation (RSV‐4′‐OH group→ PPAR*α*↑)	> 2 g orally/day→ penetrate the blood–brain barrier → cerebral spinal fluid A*β*40↓ SIRT1↑ CD147↓	[115, 116, 120]
Polycystic ovary syndrome (PCOS)	RSV: 800 mg/day for 60 days RSV: 800 mg/day for 60 days	PDE‐cAMP‐AMPK‐SIRT1 axis PI3K‐AKT signal C/EBP*β*↓SIRT1→ C/EBP*α*↓PPAR‐2↓ PCOS patients→ mtDNA copy number↑ ATP content ↑ in granulosa cells (GCs)	[151, 152]
Non‐alcoholic fatty acid disease (NAFLD) [IRCT201511233664N16]	RSV 12 weeks 300 mg/day × 2 times	RSV → supplementation in serum→plasminogen activator inhibitor‐1 (not change) adiponectin, FGF‐21 hs‐CRP (not change) RSV supplementation did not cause significant change in serum levels of liver enzymes like ALT, AST& GOT and ALP.	[153]
Dermatological diseases Wound healing Anti‐aging Psoriasis Atopic dermatitis Melanoma Acne Herpes simplex virus	500 ng/kg dose oral administration of oxy‐RSV	RSV → see various effect in human trail	[154]
Colorectal cancer [ISRCTN05926847]	COLO‐PRECENT platform RCT: aspirin (75 mg/day) Metformin (500 mg twice/day) + 5 mg RSV or 1 g RSV	High‐risk individual (BCSP) → prevention	[155]
Age‐related macular degeneration (AMD) [NCT05062486]	Oral RQC (RES, Quercetin, Curcumin) or curcumin and followed for 2 years	RQC → drusen volume↓ than curcumin only Larger drusen reduced in volume significantly	[156]
Cardiovascular diseases (CVD)	Oral hypoglycemic agents+ RSV (250 mg/day) 6 months	Randomized controlled trails (RCTs) with RSV supplementation oral hypoglycemic agent +250 mg/day RSV for 6 months→ Significantly reduction in SBP and DBP. (AMPK/Sirt1/PGC‐1↑) (Nrf2/Keep1↑ → ARE activation) RSV → gut‐microbiota driven methylases →CVD↓	[157]
Dental cancer	RSV: 100 mg/day	RSV decreased mutant *Streptococcus* and biofilm metabolic activity (50 and 200 μg/mL) RSV is significant antibiotic and antibacterial	[158]
Head and neck cancer	RSV: 400 mg/day	RSV group increased in GPX, SOD, malonaldehyde (MDA) total antioxidant capacity	[159]
Coronary artery disease	RSV (500 mg/day) with beet root extract (500 mg/day)	This combined trail improved vagal regeneration and heart rate recovery	[160]
Overweight/obesity	RSV (80 mg/day) + epigallocatechin‐3‐gallate (EGCG) (282 mg/day)	RSV+ EGCG supplementation did not affect gut microbiota composition	[161]
Knee osteoarthritis (OA)	RSV (40 mg twice/day) 7 days	Oral RSV did not reduce pain in patients with painful knee OA	[162]
Chronic kidney disease and diabetes	RSV (400 mg/day)	Endothelial function was enhanced by RSV supplementation RSV enhanced the dilation caused by flow	[163, 164]
Marfan syndrome (MFS)	RSV (500 mg/day)	Adult patients with MFS, RSV therapy for a year may stabilize the aortic development rate	[165]
Ulcerative colitis	RSV (500 mg/day) + Mediterranean diet (MD)	The additive advantage might be minimal, and main effect did not differ substantially from that of MD alone	[166]
Post‐menopausal women with insulin resistance	RSV (500 mg/day) + vitamin C	This supplementation decreases marks of oxidative stress and total antioxidant capacity (TAC) → Combined treatment can reduce protein damage more effectively than single	[167]

Thus, far current clinical trials of RSV have targeted cardiovascular diseases, cancer, diabetes, NAFLD, neurological diseases, and obesity‐related diseases. Current clinical trials have demonstrated that RSV is well tolerated and has a beneficial effect on disease‐specific biomarkers. However, RSV has sometimes had ambiguous and detrimental effects in certain cancers and NAFLD [169]. For example, the therapeutic effects of RSV depend on several factors. It was more effective in certain cancers than in others. It reduced the expression of certain breast cancer‐related genes epigenetically but caused severe adverse incidents in patients with multiple myeloma. Thus, its effects are limited to specific types of cancer. Vast preclinical data supporting RSV's chemo‐preventive and therapeutic functions thus warrant further clinical studies.

RSV was useful for patients with cardiovascular disorders, but perhaps more so in certain demographics, because it was not effective in significantly overweight individuals and detrimental in patients with schizophrenia (NCT02062190). Thus, effective RSV treatment depends on the individual disorder's symptoms. Furthermore, RSV can increase insulin sensitivity and decrease blood glucose levels, which are associated with diabetes. The effects of RSV on NAFLD remain inconclusive because half of the clinical trials found that RSV positively affected NAFLD biomarkers, while the other half observed no such changes in the same biomarkers. Similarly, clinical trials of RSV in obesity provided conflicting results. Therefore, more clinical data are needed to understand RSV's therapeutic potential, and pharmaceutical efforts should focus on developing an RSV derivative with better bioavailability. Phase I and II clinical studies in healthy human volunteers or patients with type II diabetes mellitus have started to identify possible roles for RSV as an effective dietary supplement, and the compound appears to have no harmful effects at doses up to 5 g/day.

There is also growing interest in pharmacological interventions targeting cardiovascular risk factors, such as atherosclerosis and type II diabetes, to prevent neurodegenerative diseases, such as Alzheimer's disease (National Institute on Aging, NCT01842399; 2022‐12‐23) and retinal disease [170], and coronavirus disease 2019 [150]. The efficacy of RSV in regulating platelet activation/aggregation, preventing ROS‐induced inflammation by reducing neutrophil levels, inhibiting coagulation, and increasing insulin sensitivity is well known. Thus, the use of RSV as a natural compound for treating coronavirus disease 2019 can be innovative, because most previous studies have focused on chemical drugs or vaccines.

Hypertension is recognized as a disease affecting the global population. Novel therapeutic trials that complement the current hypertension management protocols will help decrease the disease burden. RSV supplementation for a short duration enhanced nitric oxide production but did not significantly reduce blood pressure in patients with prehypertension or stage I hypertension. Nitric oxide levels were considerably higher in the RSV‐treated group than in the placebo‐treated group. This finding requires verification through larger, long‐term clinical trials [149].

Drusen are the single most significant risk factor for the development of advanced age‐related macular degeneration (AMD). The primary drusen volume was assessed in a 24‐month phase II clinical trial evaluating a combination treatment of oral RSV, quercetin, and curcumin (RQC) in patients with intermediate AMD. After 24 months, participants receiving RQC demonstrated significantly reduced drusen volume compared to those receiving only curcumin. Larger drusen exhibited greater reductions in volume. These findings indicated that RQC targeted the pathophysiological mechanisms associated with the presence of large drusen. Consequently, supplementation with RQC could reduce the risk of progression to the wet form of advanced AMD in patients with early to intermediate stage AMD [156]. Similarly, RSV can be used in conjunction with other antioxidant drugs, as described above, to efficiently affect the oxidation stage, which may be useful for future applications as therapeutic tools.

The protective effects of RSV against high‐fat diet‐induced hepatic dysfunction and intestinal mucus layer depletion with a focus on antioxidant and anti‐inflammatory mechanisms were also reported [171]. Thus, RSV therapy decreased body weight gain and adiposity, lowered plasma and hepatic oxidative stress markers, and restored endogenous antioxidant enzyme activity. Levels of liver markers, including alkaline phosphatase, alanine transferase, and aspartate aminotransferase, also returned to normal in RSV‐treated animals. These findings indicated that RSV exerted a multiorgan protective effect by simultaneously preserving intestinal mucus, improving hepatic antioxidant defenses, and reducing fibrosis. Furthermore, this study highlighted a novel gut–liver axis mechanism for RSV action that extends beyond its well‐known anti‐obesity and mitochondrial benefits.

Another new area for RSV treatment research is chronic pain. Various analgesics are employed in pain management, including opioids, acetaminophen, and nonsteroidal anti‐inflammatory drugs. However, the long‐term use of these medications is often limited by adverse effects, highlighting the urgent need for alternative natural therapies to alleviate chronic pain effectively.

In summary, RSV functions as a natural therapeutic agent, demonstrating great potential for developing new analgesic medications and providing clinical values in the treatment of chronic pain. As a choice for an alternative analgesic, additional ongoing clinical studies are required to investigate the practical use of RSV in the treatment of chronic pain [172].

In most clinical trials, the major obstacle presented was the poor bioavailability of RSV. Thus, this issue should be solved soon. Thus, preclinical studies are necessary to obtain conclusive results by evaluating the merits and demerits of each trial case.

## 11 Conclusion and Perspectives

RSV exhibited broad‐spectrum bioactivity, including anticancer, antioxidant, and neuroprotective effects. In oncology, it orchestrated tumor suppressors and pro‐apoptotic pathways while inhibiting inflammation and oxidative stress. In neurodegeneration, the pleiotropic mechanisms of RSV, such as activating SIRT, clearing protein aggregates, and maintaining redox homeostasis, suggest its therapeutic potential in both AD and PD. Moreover, RSV aids in the osteogenic and neuronal differentiation of MSCs in a concentration‐dependent manner, which is beneficial for bone and neural tissue repair. Despite these promising findings, clinical translation is hindered by its poor solubility and metabolic instability. Advances in drug delivery systems, improved patient stratification using biomarkers, and conducting large‐scale controlled trials are essential to harness RSV's full therapeutic potential. Its combination with agents such as CoQ10, as well as standard therapeutics, offers promising avenues for integrative strategies. As the field advances, RSV may serve as a central molecule in precision medicine frameworks targeting oxidative stress, inflammation, and cellular degeneration. To define precise, targeted supplementation for clinical applications, future research needs to utilize artificial intelligence and multimodal approaches to understand the intricate relationships among factors such as dietary habits, the microbiota–gut–brain axis, cancer stem cells and their microenvironments, and cellular plasticity within specific organs. The balance of oxidative stress and antioxidation is the new target of the therapeutic modulation by RSV and antioxidants to maintain the metabolism of the animals. The environmental and the genetic targets should be identified for the treatment with RSV and antioxidants to cure and maintain homeostasis.

## Author Contributions

Kenly Wuputra wrote the original draft and revised the final draft and drew the Figures and Tables. Chia‐Che Ku revised to write the original draft and drew Figures and Tables. Ying‐Chu Lin revised the original draft and drew the Tables and Figures. Yi‐Chun Tsai revised the final draft. Deng‐Chyang Wu revised the final draft. Yukio Mitsui advised the original draft and revised the final draft. Maki Satou revised the original and the final draft. Yuuki Tanaka revised the final draft. Shigeo Saito designated the original idea and developed it in detail, provided helpful feedback, wrote the original draft, drew the Figures and Tables, and revised the final draft. Kazunari K. Yokoyama developed the original idea, developed it in detail, and revised the original and final draft. All authors participated in drafting, writing, and editing the manuscript, and approving it for submission.

## Funding

This work was supported by grants from the Ministry of Science and Technology (MOST111‐2314‐B‐037‐009), the National Health Research Institutes (NHRI‐EX109‐10720SI), Kaohsiung Medical University Hospital (KMUH111‐1R77; KMUH‐DK(A)112002; KMGH‐113G008; KMGH‐113G025, KMHK‐113‐23), NYCU‐KMU Research project (NYCUKMU‐114‐1002), and Kaohsiung Medical University (KMU‐TC113A02).

## Ethics Statement

The authors have nothing to report.

## Consent

All authors agree to publish this article in BioFactors.

## Conflicts of Interest

The authors declare no conflicts of interest.

## Data Availability

The data that support the findings of this study are available on request from the corresponding author. The data are not publicly available due to privacy or ethical restrictions.
